# Supplementary material for: FERN – a Java framework for stochastic simulation and evaluation of reaction networks
Source: BMC Bioinformatics. 2008 Aug 29;9:356. doi: 10.1186/1471-2105-9-356 (PMC2553347; doi:10.1186/1471-2105-9-356)
Supplement: Additional file 1 — FERN distribution, Version 1.3. This archive contains the FERN source code and binaries as well as documentation and example models in FernML and SBML. [file 1471-2105-9-356-S1.zip › fern/doc/javadoc/fern/tools/gnuplot/package-use.html]

Uses of Package fern.tools.gnuplot


---


|  |  |  |
| --- | --- | --- |
| **Overview** \| **Package** \| Class \| **Use** \| **Tree** \| **Deprecated** \| **Index** \| **Help** | |  |
| PREV   NEXT | **FRAMES**    **NO FRAMES**     **All Classes** |  |


---


## **Uses of Package fern.tools.gnuplot**

| Packages that use fern.tools.gnuplot | |
| --- | --- |
| **fern.benchmark** | Provides classes for benchmarking simulations (time benchmarks, histogram distance calculation). |
| **fern.simulation.observer** | Provides classes to observe certain aspects of simulations. |
| **fern.tools.gnuplot** | Provides classes to use gnuplot from java. |

Classes in fern.tools.gnuplot used by fern.benchmark

****GnuPlot****
  
          The class `GnuPlot` provides methods for handling gnuplot data.

Classes in fern.tools.gnuplot used by fern.simulation.observer

****GnuPlot****
  
          The class `GnuPlot` provides methods for handling gnuplot data.

Classes in fern.tools.gnuplot used by fern.tools.gnuplot

****ArrayMatrixAxes****
  
          Implementation of `Axes` to use with array matrixes
of any number type (the type of the arrays is inferred by
using the reflection api).

****Axes****
  
          Contains the data columns for `GnuPlot`.

****GnuPlot****
  
          The class `GnuPlot` provides methods for handling gnuplot data.

---


|  |  |  |
| --- | --- | --- |
| **Overview** \| **Package** \| Class \| **Use** \| **Tree** \| **Deprecated** \| **Index** \| **Help** | |  |
| PREV   NEXT | **FRAMES**    **NO FRAMES**     **All Classes** |  |


---
